# Protective Human Leucocyte Antigen Haplotype, HLA-DRB1*01-B*14, against Chronic Chagas Disease in Bolivia

**DOI:** 10.1371/journal.pntd.0001587

**Published:** 2012-03-20

**Authors:** Florencia del Puerto, Juan Eiki Nishizawa, Mihoko Kikuchi, Yelin Roca, Cinthia Avilas, Alberto Gianella, Javier Lora, Freddy Udalrico Gutierrez Velarde, Sachio Miura, Norihiro Komiya, Koji Maemura, Kenji Hirayama

**Affiliations:** 1 Department of Immunogenetics, Institute of Tropical Medicine (NEKKEN), and Global COE Program, Nagasaki University, Nagasaki, Japan; 2 Clinica Siraní, Santa Cruz, Bolivia; 3 Center for International Collaboration Research (CICORN), Nagasaki University, Nagasaki, Japan; 4 Centro Nacional de Enfermedades Tropicales, Santa Cruz, Bolivia; 5 Hospital Universitario Japonés, Santa Cruz, Bolivia; 6 Department of Tropical Medicine and Parasitology, School of Medicine, Keio University, Tokyo, Japan; 7 Department of Cardiovascular Medicine, Nagasaki University Graduate School of Biomedical Science, Nagasaki, Japan; National Institutes of Health, United States of America

## Abstract

**Background:**

Chagas disease, caused by the flagellate parasite *Trypanosoma cruz*i affects 8–10 million people in Latin America. The mechanisms that underlie the development of complications of chronic Chagas disease, characterized primarily by pathology of the heart and digestive system, are not currently understood. To identify possible host genetic factors that may influence the clinical course of Chagas disease, Human Leucocyte Antigen (HLA) regional gene polymorphism was analyzed in patients presenting with differing clinical symptoms.

**Methodology:**

Two hundred and twenty nine chronic Chagas disease patients in Santa Cruz, Bolivia, were examined by serological tests, electrocardiogram (ECG), and Barium enema colon X-ray. 31.4% of the examinees showed ECG alterations, 15.7% megacolon and 58.1% showed neither of them. A further 62 seropositive megacolon patients who had undergone colonectomy due to acute abdomen were recruited. We analyzed their HLA genetic polymorphisms (HLA-A, HLA-B, MICA, MICB, DRB1 and TNF-alpha promoter region) mainly through Sequence based and LABType SSO typing test using LUMINEX Technology.

**Principal Findings:**

The frequencies of HLA-DRB1*01 and HLA-B*14:02 were significantly lower in patients suffering from megacolon as well as in those with ECG alteration and/or megacolon compared with a group of patients with indeterminate symptoms. The DRB1*0102, B*1402 and MICA*011 alleles were in strong Linkage Disequilibrium (LD), and the HLA-DRB1*01-B*14-MICA*011haplotype was associated with resistance against chronic Chagas disease.

**Conclusions:**

This is the first report of HLA haplotype association with resistance to chronic Chagas disease.

## Author Summary

Chronic Chagas disease consists of four different forms categorized on the basis of their clinical manifestations, namely; cardiac, digestive, cardiodigestive and indeterminate. In Latin America, there are 8–10 million seropositive persons who are at risk of, or have already developed serious clinical complications and who have limited access to effective treatment. The cardiac and digestive forms are characterized by tissue damage caused by persistent infection of *Trypanosoma cruzi* and are thought to be modulated by host immunity. In our large scale screening for chronic Chagas disease in Santa Cruz, Bolivia, hearts and colons of 229 seropositive patients were examined. We found 31.4% of patients had abnormal electrocardiograms (ECGs), 15.7% presented with megacolon, 5.2% had a combination of abnormal ECG and megacolon, and 58.1% were of indeterminate status. Previously, we attempted to ascertain whether parasite genetic polymorphism might account for the differences in clinical manefestations, by analyzing parasite DNA taken from the same study group (with the addition of a further 62 megacolon post-operational patients). We found no relationships between parasite lineages and clinical disease form. The present study reveals that host HLA polymorphisms associate with clinical manifestations of Chagas.

## Introduction

Following an extensive control program consisting of vector control, serological screening in blood banks and identification and treatment of congenital transmission, the estimated number of people infected with *Trypanosoma cruzi*, the causal agent of Chagas disease in Latin America, has fallen from approximately 20 million in 1981 to around 8–10 million in 2009 [1], [2]. Most of the seropositive patients are chronically infected and more than 10,000 deaths are estimated to occur annually from the disease [1], [3], [4].

Cardiac myositis and autonomous neuroplexus degeneration of the digestive tract are major histopathological alterations that can arise during Chagas disease, and may lead to cardiac failure, digestive abnormalities, megacolon or megaesophagus. Based on these pathologies, there are often considered to be three major clinical forms of Chagas disease; cardiac, digestive and indeterminate [5], [6]. This variation in pathological manifestation has been reported to relate to differences in host immune response, such as the ability to control parasitaemia, the strength of inflammatory reactions, and the induction of autoimmune like responses [7]–[11]. Indeterminate phase T-cells have been reported to correspond with modulatory responses such as increased IL-10 production by CD4^+^CD28^−^ T cells and the expression of CTLA-4, a ligand that is involved in modulation of T-cell responses by CD8^+^ T-cells [12], [13]. Additionally, the unregulated production of IFN-gamma by CD8^+^ T-cells in cardiac Chagas disease patients has been reported, which might result in the destruction of heart tissue due to its cytotoxic effect [12].

The highly polymorphic HLA Class I and II molecules determine the efficiency of *T. cruzi* epitope presentation to T lymphocytes that could affect the clinical course of Chagas disease [14], [15]. Several HLA alleles and haplotypes have been reported to be associated with Cardiac Chagas disease in Chile, Venezuela, Brazil and Guatemala [16]–[18]. The HLA region contains not only classical HLA genes but a wide variety of immunologically relevant genes, such as nonclassical class I genes (MICA, MICB) [19], [20], and class III genes (TNF-alpha, beta), that may be involved in pathogenesis [21], [22].

In the present study, we investigated HLA class I (A, B, MICA, MICB), Class II (DRB1) and Class III (TNF-alpha) gene polymorphism in seropositive chronic Chagas patients in Bolivia, characterized by electrocardiogram (ECG), barium enema colon X-ray examinations and/or surgical operation.

## Materials and Methods

### Patients

The study subjects were described previously [23]. Two hundred and ninety one patients with chronic Chagas disease (136 men and 155 women, mean age 45 years) were recruited from the Centro Nacional de Enfermedades Tropicales (CENETROP) (91 men and 119 women), Hospital Primero de Mayo (12 men and 7 women) and from post-operative patients at the Hospital Universitario Japonés (HUJ) (33 men and 29 women) in Santa Cruz, Bolivia.

Upon medical examination of patients other than the HUJ patients, if serological tests (Indirect Haemaglutination assay (IHA) and Indirect Immunofluorescence test (IIF) [3]) were positive, they were asked to participate in the study and signed informed consent was obtained. Informed consent was also obtained for the HUJ post-operative patients, using the same protocol.

ECG abnormalities were diagnosed based on the Minnesota Code Criteria and were confirmed independently by two cardiologists. Colon X-ray with barium enema examination was performed for the detection of megacolon. To exclude the possibility of including individuals who were asymptomatic upon examination, but who may not have had adequate time post-infection for symptoms to become apparent, those under 30 years of age were excluded.

Finally, 229 seropositive Chagas outpatients in Santa Cruz, Bolivia, were examined by ECG and/or barium enema colon X-ray as shown in Table 1. The 62 post-operational patients from HUJ were confirmed to be suffering from Chagas megacolon during the admission period.

**Table 1:** Subjects.

Grouping Criteria	IIFζ	HAAϕ	Mega colon*	ECG alteration**	N = 291	Male/Female	Age ±SD
Indeterminate	+	+	−	−	70	31/39	43±8.5
Megacolon	+	+	+	−	36	17/19	47±12.0
Megacolon	+	+	+	NE	45	26/19	56±16.6
ECG alteration	+	+	−	+	40	20/20	39±9.8
ECG alteration	+	+	NE	+	20	10/10	41±10.6
Megacolon and ECG alteration	+	+	+	+	17	6/11	48±14.1
Not determined	+	+	NE	−	36	15/21	42±8.8
Not determined	+	+	−	NE	27	11/16	43±7.5

*: Examination of Colon by Barium enema and X-ray: +; Megacolon, −; no abnormality, NE; not examined.

**: Examination of Electrocardiogram. +; typical alterations, −; no alteration, NE; not examined.

**ζ:** Indirect Immunofluorescence Test.

**ϕ:** Haemaglutination Assay.

The experimental protocol was approved by the Institutional Ethical Review Committee of the Institute of Tropical Medicine, Nagasaki University (No. 0210170018) and the Centro Nacional de Enfermedades Tropicales (CENETROP).

### DNA extraction

Genomic DNA was extracted from 10 mL of whole blood containing 10 mM EDTA using a DNA extraction kit (QIAGEN GmbH, FRG) and was stored at −20°C until use.

### Typing of MICA, MICB, TNF alpha promoter region and GCT repeat polymorphism in the MICA gene transmembrane region (MICA-TM)

MICA, MICB, TNF-alpha promoter region and MICA-TM typing was performed as previously described [19]–[21]. Sequences were obtained from a 3730 DNA Analyzer (Applied Biosystems, USA) and submitted to the Assign- software ATF (Conexio Genomics ATF, Australia) for allele identification. The TNFP alleles were determined according to Higuchi T *et al* (1998) and Ubalee R *et al* (2001) [21], [22].

MICA-TM alleles were typed by sequencing on a 3730 DNA analyzer (Applied Biosstems, USA) and analyzed with the GeneMapper Software Version 3.7 (Applied Biosystems, USA). The fluorescent primers, 5′ F (GCC CAG TGT ATA ACA AGT CCC-6-FAM) and 3′ R (CCT TAC CAT CTC CAG AAA CTG C) were used.

### HLA-A, HLA-B, HLA-DRB1 typing

Typing was carried out according to the manufacturer's specification for LABType SSO Typing, testing for each locus using LUMINEX Technology (ONE LAMBDA, INC, USA) and the retrieved output was analyzed by HLA Fusion software (ONE LAMBDA, INC, USA) for allele identification.

### Statistical analysis

Statistical analysis was performed at the two and four digits levels. Allele frequencies less than 5% were removed from the analysis. The statistical significance and odds ratio (OR) of allele frequencies between each group was determined by Chi square and Fisher's exact tests using the StatsDirec software (StatsDirect Ltd, UK). *P*-values were considered significant when <0.05 following Bonferoni correction (Pc). Hardy-Weinberg Equilibrium, linkage disequilibrium (LD) and Haplotype analyses were calculated with PyPopWin32.0.7.0 software [24].

## Results

### Frequencies of HLA-B*14 and HLA-DRB1*01 significantly decreased in megacolon patients compared with indeterminate patients

In the two digits analysis (Table S1, S3, S5), we observed a significant decreased frequency of alleles DRB1*01 and B*14 in the megacolon patients compared with indeterminate patients (Table 2). The frequency of HLA-B*14 was also significantly lower in the patients with ECG alteration compared to the indeterminate patients. In the four digits analysis (Table S2, S4, S6), DRB1*01:01, *01:02 and B*14:02 had the same tendency towards lower allele frequencies in the megacolon patients compared with the indeterminate patients. The frequency of the HLA-B*14:02 allele was significantly lower in the ECG alteration and/or megacolon patients compared with those of indeterminate symptoms (Table 2). The frequency of MICA*011 was also significantly lower in the complication positive groups (Table 2, S7).

**Table 2:** Two-digit and four-digit alleles association with Clinical Manifestations of Chagas disease.

HLA locus	Indeterminate (N = 70)		Megacolon (N = 98)		ECG Alteration (N = 77)		ECG alteration and/or Megacolon (N = 158)				
HLA-DRB1*	n	(%)	n	(%)	n	(%)	n	(%)	Pv	Pc	OR [95%CI]
01	12	(17.1)	**1**	**(1.0)**	9	(11.7)	10	(6.3)	0.0001*	0.001*	0.049 [0.001–0.358]*
01:01	4	(5.7)	0	(0.0)	2	(2.6)	2	(1.3)			
01:02	8	(11.4)	**1**	**(1.0)**	3	(3.9)	**4**	**(2.5)**	0.011*0.009ζ	NS*NSζ	0.089 [0.002–0.687] *0.200 [0.043–0.790]
01:03	1	(1.4)	0	(0.0)	4	(5.2)	4	(2.5)			
03:01ϕ	4	(5.7)	10	(10.2)	3	(3.9)	13	(8.2)			

HLA-A*	n	(%)	n	(%)	n	(%)	n	(%)	Pv	Pc	OR [95%CI]
01	10	(14.3)	**4**	**(4.1)**	16	(20.8)	20	(12.7)	0.024*	NS*	0.250 [0.056–0.943]*
01:01ϕ	4	(5.7)	3	(3.1)	**14**	**(18.2)**	17	(10.8)	0.024**	NS**	3.667 [1.067–15.993]**
01:06	4	(5.7)	1	(1.0)	1	(1.3)	1	(0.6)			
01:07	0	(0.0)	0	(0.0)	0	(0.0)	1	(0.6)			
01:14	2	(2.9)	0	(0.0)	1	(1.3)	0	(0.0)			
33:01	1	(1.4)	5	(5.1)	1	(1.3)	6	(3.8)			

HLA-B*	n	(%)	n	(%)	n	(%)	n	(%)	Pv	Pc	OR [95%CI]
14	10	(14.3)	**2**	**(2.0)**	**3**	**(3.9)**	**5**	**(3.2)**	0.004*0.040**0.003ζ	0.049*NS**0.040ζ	0.125 [0.013–0.623]*0.245 [0.042–1.010]**0.196 [0.051–0.666]ζ
14:01	1	(1.4)	0	(0.0)	2	(2.6)	2	(1.3)			
14:02	8	(11.4)	**2**	**(2.0)**	**1**	**(1.3)**	**3**	**(1.9)**	0.018*0.014**0.004ζ	NS*NS**0.049ζ	0.161 [0.016–0.854]*0.103 [0.002–0.805]**0.149 [0.025–0.675]ζ
14:06	1	(1.4)	0	(0.0)	0	(0.0)	0	(0.0)			
08:01ϕ	4	(5.7)	6	(6.1)	5	(6.5)	11	(7.0)			

MICA*	n	(%)	n	(%)	n	(%)	n	(%)	Pv	Pc	OR [95%CI]
011	6	(8.6)	**0**	**(0.0)**	1	(1.3)	**1**	**(0.6)**	0.005*0.004ζ	NS*0.048ζ	0.050 [0.000–0.832]*0.069 [0.001–0.584]ζ
034	1	(1.4)	4	(4.1)	2	(2.6)	6	(3.8)			

MICB*	n	(%)	n	(%)	n	(%)	n	(%)	Pv	Pc	OR [95%CI]
008ϕ	9	(12.9)	5	(5.1)	9	(11.7)	14	(8.9)			

Footnote for Table 2.

*: Comparison between Megacolon vs Indeterminate.

**: Comparison between ECG alteration vs Indeterminate.

**ζ:** Comparison between (ECG+ &/or Megacolon+) vs Indeterminate.

**ϕ:** Linkage Disequilibrium group of HLA-DRB1*03:01-MICB*008-B*08:01-A*01:01 as shown in Table 3.

### TNF-alpha promoter region polymorphism and MICB alleles

None of the TNF-*alpha* promoter region and MICB (Table S11 and S8) alleles tested here were significantly associated with the clinical groups compared with the indeterminate group.

### Linkage disequilibrium (LD) between the alleles of HLA-DRB1, A, B, and MICA, B loci

Linkage disequilibrium (LD) was calculated for all combinations of alleles (Table S1, Table S2, Table S3, Table S4, Table S5, Table S6, Table S7, Table S8, Table S9), and significantly strong LDs was observed between the alleles, HLA-DRB1*01, A*33:01, B*14:02 and MICA*011 as shown in Table 3. Except for HLA-A*33:01, all the linkage group alleles showed the same tendency of being associated with protection against megacolon (Table 2). HLA-A*01:01 showed strong LD with HLA-B*08:01, DRB1*0301 and MICB*008. Although HLA-A*01:01 showed a non-significant tendency of association with non-megacolon patients, none of the LD group alleles showed any similar tendency (Table 2). No LD was observed between any HLA alleles and TNF-alpha promoter haplotypes.

**Table 3:** Linkage analysis of the alleles with positive association to the complication of Chagas.

Allele 1	Allele 2	observed	expected	diseq	chisq	Degree of freedom	P value
HLA-DRB1*							
01:02	A*33:01	4	0.27	0.006	53.81	20	<0.0001
01:02	B*14:02	8	1.71	0.011	26.81	16	<0.05
01:02	MICA*011	6	0.25	0.009	124.92	28	<0.0001
HLA-A*							
01:01	DRB1*03:01	8	1.01	0.012	51.79	20	<0.0005
01:01	B*08:01	10	0.82	0.016	111.62	20	<0.0001
01:01	MICB*008	8	1.23	0.011	40.29	25	<0.05
HLA-B*							
14:02	DRB1*01:02	8	1.71	0.011	26.81	16	<0.05
14:02	MICA*034	4	0.21	0.007	69.09	28	<0.0001
14:02	MICA*011	8	0.24	0.013	239.21	28	<0.0001
MICA*							
011	B*14:02	8	0.24	0.013	239.21	28	<0.0001
011	DRB1*01:02	6	0.25	0.009	124.92	28	<0.0001
MICB*							
008	A*01:01	8	1.23	0.011	40.29	25	<0.05
008	B*08:01	10	1.11	0.015	73.57	20	<0.0001
008	DRB1*03:01	8	1.25	0.011	36.12	20	<0.05

## Discussion

We have previously shown that there was no association between *T. cruzi* lineage or sub lineage and the clinical manifestation of Chagas disease in samples from Bolivia [23]. Here, we have analysed the same samples in order to determine if there were any associations between clinical disease and host genetic variation. We analysed polymorphic genes located in the MHC region, consisting of three sub regions; class I, II and III. Human MHC, HLA class I and II molecules play a crucial role in determining individual acquired immune responsiveness through the presentation of pathogen-derived peptides to CD8^+^ and CD4^+^ T-cells. In the class III sub region, there are a variety of genes related to immunity such as complement, TNF-alpha, Lymphotoxin, *etc.* [24].

Our study revealed that the HLA-DRB1*01-B*14-MICA*011 haplotype was significantly associated with protection from Chagasic megacolon. As the linkage disequilibrium between the HLA-B*14 and DRB1*01 was strong (Table 3), it was difficult to determine the primary associated locus within the haplotype.

HLA-class I is the antigen-presenting molecule on the cell-surface of infected host cells which stimulates microbe-derived antigen specific CD8^+^ T-cells. Therefore, HLA-B*14 itself could be directly related to protective T-cell immunity as was suggested by the Chagas disease mouse model [25].

If HLA-B*14 is more efficient at stimulating protective T-cells through the binding of antigenic peptides, other HLA-class I molecules that share the same antigen binding motif should show the same protective association. HLA-B*14 consists of three 4-digit alleles, HLAB*14:01, 14:02 and 14:06 that share the same antigen binding motifs but the latter two alleles are so rare that it was impossible to analyse their effect. As the HLA-B*14 alleles belong to the B27 supertype group that share the same anchoring residues in the peptide binding groove, we analyzed the member alleles B*38:01, 39:04, 39:05. 39:06, 39:14, 48:01, 48:03 for their total effect on protection against complication, and found no association (Tables S6 and S10) [26]. This finding did not, therefore, support the hypothesis that the association between HLA-B*14 and protection against clinical Chagas disease is driven by the ability of the gene product to more effectively stimulate protective T-cells than other alleles of this gene.

The peripheral human and mice CD8^+^T-cells reactive to *Trypanosome* antigens have been identified [27]. Interestingly, HLA-A*02:01 restricted epitopes from cruzipain and FL-160 were frequently recognized by PBMC of patients with Cardiopathy [28]. Moreover, an HLA-A2 tetramer experiment showed that the number of IFN-gamma producing amastigote-specific CD8^+^ T-cells inversely correlated with the severity of the disease [29]. It will be interesting to see if the same phenomenon occurred in the HLA-B*14 patients.

Immuno-regulatory mechanisms have been reported to be associated with clinical forms including Treg cells [30], NKT cells and NK cells [31]. Whereas the HLA involvement in the induction of Treg cells is not yet clear, HLA-class I can interact with NK cells to suppress their activity through various inhibitory receptors such as KIR. HLA-B*14 belongs to the Bw6 family, the members of which preferentially stimulate specific members of the KIR family [32], [33], which could regulate NK cell activity during inflammation. HLA-non classical class I, MICA*011, which was closely linked to HLA-B*14 and DRB1*01 might also be functional as MICA is known to stimulate gamma-delta T-cells in the gut mucosa; a phenomenon that could relate to megacolon [34].

HLA-class II can present antigen to CD4^+^ T-cells so HLA-DRB1*01 may also be directly involved in the pathogenesis as well as HLA-B*14. Many autoimmune diseases are reported to be associated with specific HLA-class II alleles [35]. Auto-reactive processes that involve the activation of cytokine producing T-cells may occur during infection. As was previously suggested, autoimmune mechanisms in the pathogenesis of chronic Chagas heart and colon may be regulated by the HLA-class II.

We analyzed 4-digit HLA-DRB1 alleles for association with Chagas disease clinical manifestations. As shown in Table 2, the HLA-DRB1*01 group included three alleles, DRB1*01:01, 01:02, 01:03 and all of them showed the same protective tendency when compared between the megacolon and indeterminate symptom groups. As two of them, 01:01 and 01:02 shared the same peptide-binding motif [36], we considered that the HLA-DR molecule itself was functionally related to resistance to megacolon. It was previously reported that the DRB1*01 allele was associated with susceptibility to Chagas cardiomyopathy in Venezuela [37]. This opposite association to the megacolon resistance observed in the present work requires further clarification.

Despite the strong LD shown within the HLA-DRB1*01-B*14 haplotype, there was no strong linkage between TNF-alpha promoter alleles that may influence the levels of its production by immune cells [21]. However, between HLA-DRB1 and HLA-B loci, 1270 kb of class III sub region containing more than 60 genes such as complements, heat shock proteins, 21-hydroxylase, are present that might be relevant to pathogenesis. The HLA-B*14:02-DRB1*01:02 haplotype was reported to be associated with V281L polymorphism in 21-hydroxylase in African-American and Caucasian populations [38]. The same kind of abnormality was also reported in the HLA-A*01:01-B*08-DRB1*03 haplotype that is associated with several diseases such as allergy and viral infectious diseases [39]. It was not significantly associated with Chagas. However, HLA-A*01:01 also showed a decreased frequency in the megacolon patients. Whole sequencing of the class III region of the associated haplotypes would be the next target for clarification of any genetic resistance.

We have no information relating to the immunological characteristics of the individuals who poscessed those haplotypes that might be associated with lymphocyte activation during a chronic infection. We did, however, analyze the relationship between individuals' specific antibody titers and their HLA alleles (data not shown) but there was no clear correlation. About 7.1% of the seropositive indeterminate individuals were estimated to carry this haplotype; therefore further to identify its characteristic immunological function is feasible. To our knowledge, this is the first report of resistant HLA haplotype association with chronic Chagas diagnosed by the active examination of silent colon lesion.

## Supporting Information

Table S1 **The frequency of the Alleles of HLA-DRB1 locus. Two digits analysis.**
(DOC)

Table S2 **The frequency of the Alleles of HLA-DRB1 locus. Four digits analysis.**
(DOC)

Table S3 **The frequency of the Alleles of HLA-A locus. Two digits analysis.**
(DOC)

Table S4 **The frequency of the Alleles of HLA-A locus. Four digits analysis.**
(DOC)

Table S5 **The frequency of the Alleles of HLA-B locus. Two digits analysis.**
(DOC)

Table S6 **The frequency of the Alleles of HLA-B locus. Four digits analysis.**
(DOC)

Table S7 **The frequency of the Alleles of MICA locus.**
(DOC)

Table S8 **The frequency of the Alleles of MICB locus.**
(DOC)

Table S9 **The frequency of GCT triplet polymorphism in the MICA-transmembrane region.**
(DOC)

Table S10 **The frequency of B Supertype.**
(DOC)

Table S11 **The frequency of TNF-alpha promoter region polymorphism.**
(DOC)
