# Supplementary material for: Protective Human Leucocyte Antigen Haplotype, HLA-DRB1*01-B*14, against Chronic Chagas Disease in Bolivia
Source: PLoS Negl Trop Dis. 2012 Mar 20;6(3):e1587. doi: 10.1371/journal.pntd.0001587 (PMC3308929; doi:10.1371/journal.pntd.0001587)
Supplement: Table S1 — The frequency of the Alleles of HLA-DRB1 locus. Two digits analysis. (DOC) [file pntd.0001587.s001.doc]

**Table S1**. The frequency of the Alleles of HLA-DRB1 locus. Two digits analysis

|  | **Indeterminate**  **(N=70)** | | **Megacolon**  **(N=98)** | | **ECG**  **Alteration**  **(N=77)** | | **ECG alteration and/or Megacolon (N=158)** | |
| --- | --- | --- | --- | --- | --- | --- | --- | --- |
|  | n | (%) | n | (%) | n | (%) | n | (%) |
| DRB1*01 | 12 | (17.1) | 1 | (1.0) | 9 | (11.7) | 10 | (6.3) |
| DRB1*03 | 7 | (10.0) | 10 | (10.2) | 5 | (6.5) | 15 | (9.5) |
| DRB1*04 | 21 | (30.0) | 37 | (37.8) | 28 | (36.4) | 55 | (34.8) |
| DRB1*07 | 6 | (8.6) | 6 | (6.1) | 10 | (13.0) | 14 | (8.9) |
| DRB1*08 | 12 | (17.1) | 32 | (32.7) | 22 | (28.6) | 50 | (31.6) |
| DRB1*09 | 11 | (15.7) | 13 | (13.3) | 11 | (14.3) | 23 | (14.6) |
| DRB1*11 | 8 | (11.4) | 7 | (7.1) | 5 | (6.5) | 11 | (7.0) |
| DRB1*13 | 14 | (20.0) | 12 | (12.2) | 14 | (18.2) | 25 | (15.8) |
| DRB1*14 | 21 | (30.0) | 34 | (34.7) | 22 | (28.6) | 48 | (30.4) |
| DRB1*15 | 4 | (5.7) | 14 | (14.3) | 2 | (2.6) | 16 | (10.1) |
| DRB1*16 | 11 | (15.7) | 15 | (15.3) | 6 | (7.8) | 21 | (13.3) |
