# Supplementary material for: Protective Human Leucocyte Antigen Haplotype, HLA-DRB1*01-B*14, against Chronic Chagas Disease in Bolivia
Source: PLoS Negl Trop Dis. 2012 Mar 20;6(3):e1587. doi: 10.1371/journal.pntd.0001587 (PMC3308929; doi:10.1371/journal.pntd.0001587)
Supplement: Table S2 — The frequency of the Alleles of HLA-DRB1 locus. Four digits analysis. (DOC) [file pntd.0001587.s002.doc]

**Table S2**. The frequency of the Alleles of HLA-DRB1 locus. Four digits analysis

|  | **Indeterminate**  **(N=70)** | | **Megacolon**  **(N=98)** | | | **ECG**  **Alteration**  **(N=77)** | | **ECG alteration and/or Megacolon (N=158)** | | |
| --- | --- | --- | --- | --- | --- | --- | --- | --- | --- | --- |
|  | n | (%) | n | (%) | n | | (%) | | n | (%) |
| DRB1*01:01 | 4 | (5.7) | 0 | (0.0) | 2 | | (2.6) | | 2 | (1.3) |
| DRB1*01:02 | 8 | (11.4) | 1 | (1.0) | 3 | | (3.9) | | 4 | (2.5) |
| DRB1*01:03 | 1 | (1.4) | 0 | (0.0) | 4 | | (5.2) | | 4 | (2.5) |
| DRB1*03:01 | 4 | (5.7) | 10 | (10.2) | 3 | | (3.9) | | 13 | (8.2) |
| DRB1*03:05 | 1 | (1.4) | 0 | (0.0) | 1 | | (1.3) | | 1 | (0.6) |
| DRB1*03:08 | 1 | (1.4) | 0 | (0.0) | 1 | | (1.3) | | 1 | (0.6) |
| DRB1*03:20 | 1 | (1.4) | 0 | (0.0) | 0 | | (0.0) | | 0 | (0.0) |
| DRB1*04:01 | 0 | (0.0) | 1 | (1.0) | 2 | | (2.6) | | 2 | (1.3) |
| DRB1*04:02 | 0 | (0.0) | 2 | (2.0) | 1 | | (1.3) | | 3 | (1.9) |
| DRB1*04:03 | 2 | (2.9) | 1 | (1.0) | 2 | | (2.6) | | 2 | (1.3) |
| DRB1*04:04 | 9 | (12.9) | 16 | (16.3) | 11 | | (14.3) | | 24 | (15.2) |
| DRB1*04:05 | 1 | (1.4) | 6 | (6.1) | 4 | | (5.2) | | 7 | (4.4) |
| DRB1*04:07 | 6 | (8.6) | 5 | (5.1) | 4 | | (5.2) | | 8 | (5.1) |
| DRB1*04:08 | 0 | (0.0) | 0 | (0.0) | 1 | | (1.3) | | 1 | (0.6) |
| DRB1*04:10 | 0 | (0.0) | 2 | (2.0) | 3 | | (3.9) | | 5 | (3.2) |
| DRB1*04:11 | 0 | (0.0) | 1 | (1.0) | 1 | | (1.3) | | 2 | (1.3) |
| DRB1*04:14 | 1 | (1.4) | 0 | (0.0) | 0 | | (0.0) | | 0 | (0.0) |
| DRB1*04:17 | 0 | (0.0) | 1 | (1.0) | 1 | | (1.3) | | 1 | (0.6) |
| DRB1*04:35 | 1 | (1.4) | 0 | (0.0) | 0 | | (0.0) | | 0 | (0.0) |
| DRB1*04:42 | 1 | (1.4) | 0 | (0.0) | 0 | | (0.0) | | 0 | (0.0) |
| DRB1*04:43 | 1 | (1.4) | 0 | (0.0) | 0 | | (0.0) | | 0 | (0.0) |
| DRB1*04:54 | 0 | (0.0) | 2 | (2.0) | 1 | | (1.3) | | 2 | (1.3) |
| DRB1*04:77 | 0 | (0.0) | 1 | (1.0) | 0 | | (0.0) | | 1 | (0.6) |
| DRB1*07:01 | 6 | (8.6) | 6 | (6.1) | 10 | | (13.0) | | 14 | (8.9) |
| DRB1*08:01 | 0 | (0.0) | 0 | (0.0) | 1 | | (1.3) | | 1 | (0.6) |
| DRB1*08:02 | 11 | (15.7) | 30 | (30.6) | 21 | | (27.3) | | 47 | (29.7) |
| DRB1*08:04 | 1 | (1.4) | 1 | (1.0) | 0 | | (0.0) | | 1 | (0.6) |
| DRB1*08:07 | 0 | (0.0) | 1 | (1.0) | 0 | | (0.0) | | 1 | (0.6) |
| DRB1*08:09 | 0 | (0.0) | 1 | (1.0) | 0 | | (0.0) | | 1 | (0.6) |
| DRB1*09:01 | 11 | (15.7) | 13 | (13.3) | 11 | | (14.3) | | 23 | (14.6) |
| DRB1*10:01 | 0 | (0.0) | 0 | (0.0) | 2 | | (2.6) | | 2 | (1.3) |
| DRB1*10:42 | 0 | (0.0) | 1 | (1.0) | 0 | | (0.0) | | 1 | (0.6) |
| DRB1*11:01 | 2 | (2.9) | 5 | (5.1) | 3 | | (3.9) | | 8 | (5.1) |
| DRB1*11:02 | 1 | (1.4) | 0 | (0.0) | 0 | | (0.0) | | 0 | (0.0) |
| DRB1*11:03 | 0 | (0.0) | 1 | (1.0) | 1 | | (1.3) | | 1 | (0.6) |
| DRB1*11:04 | 4 | (5.7) | 1 | (1.0) | 1 | | (1.3) | | 2 | (1.3) |
| DRB1*11:68 | 1 | (1.4) | 0 | (0.0) | 0 | | (0.0) | | 0 | (0.0) |
| DRB1*12:01 | 0 | (0.0) | 0 | (0.0) | 2 | | (2.6) | | 2 | (1.3) |
| DRB1*12:14 | 0 | (0.0) | 1 | (1.0) | 1 | | (1.3) | | 1 | (0.6) |
| DRB1*13:01 | 4 | (5.7) | 4 | (4.1) | 4 | | (5.2) | | 8 | (5.1) |
| DRB1*13:02 | 3 | (4.3) | 5 | (5.1) | 6 | | (7.8) | | 11 | (7.0) |
| DRB1*13:03 | 2 | (2.9) | 0 | (0.0) | 0 | | (0.0) | | 0 | (0.0) |
| DRB1*13:04 | 1 | (1.4) | 1 | (1.0) | 0 | | (0.0) | | 1 | (0.6) |
| DRB1*13:05 | 1 | (1.4) | 0 | (0.0) | 1 | | (1.3) | | 1 | (0.6) |
| DRB1*13:27 | 1 | (1.4) | 0 | (0.0) | 1 | | (1.3) | | 1 | (0.6) |
| DRB1*13:40 | 1 | (1.4) | 0 | (0.0) | 0 | | (0.0) | | 0 | (0.0) |
| DRB1*13:41 | 0 | (0.0) | 0 | (0.0) | 1 | | (1.3) | | 1 | (0.6) |
| DRB1*13:44 | 1 | (1.4) | 0 | (0.0) | 0 | | (0.0) | | 0 | (0.0) |
| DRB1*13:47 | 1 | (1.4) | 2 | (2.0) | 3 | | (3.9) | | 4 | (2.5) |
| DRB1*13:53 | 1 | (1.4) | 0 | (0.0) | 0 | | (0.0) | | 0 | (0.0) |
| DRB1*14:01 | 1 | (1.4) | 2 | (2.0) | 1 | | (1.3) | | 3 | (1.9) |
| DRB1*14:02 | 13 | (18.6) | 23 | (23.5) | 17 | | (22.1) | | 34 | (21.5) |
| DRB1*14:06 | 5 | (7.1) | 5 | (5.1) | 1 | | (1.3) | | 6 | (3.8) |
| DRB1*14:09 | 1 | (1.4) | 0 | (0.0) | 1 | | (1.3) | | 1 | (0.6) |
| DRB1*14:27 | 0 | (0.0) | 1 | (1.0) | 1 | | (1.3) | | 1 | (0.6) |
| DRB1*14:30 | 1 | (1.4) | 0 | (0.0) | 0 | | (0.0) | | 0 | (0.0) |
| DRB1*14:48 | 1 | (1.4) | 1 | (1.0) | 0 | | (0.0) | | 1 | (0.6) |
| DRB1*14:51 | 1 | (1.4) | 2 | (2.0) | 1 | | (1.3) | | 2 | (1.3) |
| DRB1*14:52 | 0 | (0.0) | 1 | (1.0) | 1 | | (1.3) | | 1 | (0.6) |
| DRB1*15:01 | 3 | (4.3) | 11 | (11.2) | 1 | | (1.3) | | 12 | (7.6) |
| DRB1*15:02 | 1 | (1.4) | 2 | (2.0) | 1 | | (1.3) | | 3 | (1.9) |
| DRB1*15:03 | 0 | (0.0) | 1 | (1.0) | 0 | | (0.0) | | 1 | (0.6) |
| DRB1*16:01 | 2 | (2.9) | 0 | (0.0) | 1 | | (1.3) | | 1 | (0.6) |
| DRB1*16:02 | 9 | (12.9) | 15 | (15.3) | 5 | | (6.5) | | 20 | (12.7) |
