# Supplementary material for: Protective Human Leucocyte Antigen Haplotype, HLA-DRB1*01-B*14, against Chronic Chagas Disease in Bolivia
Source: PLoS Negl Trop Dis. 2012 Mar 20;6(3):e1587. doi: 10.1371/journal.pntd.0001587 (PMC3308929; doi:10.1371/journal.pntd.0001587)
Supplement: Table S3 — The frequency of the Alleles of HLA-A locus. Two digits analysis. (DOC) [file pntd.0001587.s003.doc]

**Table S3.** The frequency of the Alleles of HLA-A locus. Two digits analysis

|  | **Indeterminate**  **(N=70)** | | **Megacolon**  **(N=98)** | | **ECG**  **Alteration**  **(N=77)** | | **ECG alteration and/or Megacolon (N=158)** | |
| --- | --- | --- | --- | --- | --- | --- | --- | --- |
|  | n | (%) | n | (%) | n | (%) | n | (%) |
| **A***01 | 10 | (14.3) | 4 | (4.1) | 16 | (20.8) | 20 | (12.7) |
| **A***02 | 34 | (48.6) | 63 | (64.3) | 50 | (64.9) | 100 | (63.3) |
| **A***03 | 6 | (8.6) | 9 | (9.2) | 3 | (3.9) | 12 | (7.6) |
| **A***11 | 3 | (4.3) | 9 | (9.2) | 2 | (2.6) | 10 | (6.3) |
| **A***23 | 3 | (4.3) | 2 | (2.0) | 6 | (7.8) | 7 | (4.4) |
| **A***24 | 27 | (38.6) | 29 | (29.6) | 20 | (26.0) | 44 | (27.8) |
| **A***25 | 0 | (0.0) | 1 | (1.0) | 0 | (0.0) | 1 | (0.6) |
| **A***26 | 2 | (2.9) | 7 | (7.1) | 2 | (2.6) | 8 | (5.1) |
| **A***29 | 4 | (5.7) | 8 | (8.2) | 5 | (6.5) | 12 | (7.6) |
| **A***30 | 6 | (8.6) | 9 | (9.2) | 6 | (7.8) | 13 | (8.2) |
| **A***31 | 8 | (11.4) | 13 | (13.3) | 14 | (18.2) | 23 | (14.6) |
| **A***32 | 0 | (0.0) | 0 | (0.0) | 1 | (1.3) | 1 | (0.6) |
| **A***33 | 1 | (1.4) | 5 | (5.1) | 1 | (1.3) | 6 | (3.8) |
| **A***34 | 2 | (2.9) | 0 | (0.0) | 0 | (0.0) | 0 | (0.0) |
| **A***66 | 0 | (0.0) | 0 | (0.0) | 1 | (1.3) | 1 | (0.6) |
| **A***68 | 27 | (38.6) | 29 | (29.6) | 17 | (22.1) | 41 | (25.9) |
| **A***74 | 1 | (1.4) | 0 | (0.0) | 1 | (1.3) | 1 | (0.6) |
