# Supplementary material for: Protective Human Leucocyte Antigen Haplotype, HLA-DRB1*01-B*14, against Chronic Chagas Disease in Bolivia
Source: PLoS Negl Trop Dis. 2012 Mar 20;6(3):e1587. doi: 10.1371/journal.pntd.0001587 (PMC3308929; doi:10.1371/journal.pntd.0001587)
Supplement: Table S4 — The frequency of the Alleles of HLA-A locus. Four digits analysis. (DOC) [file pntd.0001587.s004.doc]

**Table S4.** The frequency of the Alleles of HLA-A locus. Four digits analysis

|  | **Indeterminate**  **(N=70)** | | **Megacolon**  **(N=98)** | | **ECG**  **Alteration**  **(N=77)** | | **ECG alteration and/or Megacolon (N=158)** | |
| --- | --- | --- | --- | --- | --- | --- | --- | --- |
|  | n | (%) | n | (%) | n | (%) | n | (%) |
| A*01:01 | 4 | (5.7) | 3 | (3.1) | 14 | (18.2) | 17 | (10.8) |
| A*01:06 | 4 | (5.7) | 0 | (0.0) | 1 | (1.3) | 1 | (0.6) |
| A*01:07 | 0 | (0.0) | 1 | (1.0) | 0 | (0.0) | 1 | (0.6) |
| A*01:14 | 2 | (2.9) | 0 | (0.0) | 1 | (1.3) | 1 | (0.6) |
| A*02:01 | 21 | (30.0) | 39 | (39.8) | 30 | (39.0) | 61 | (38.6) |
| A*02:02 | 2 | (2.9) | 0 | (0.0) | 0 | (0.0) | 0 | (0.0) |
| A*02:05 | 0 | (0.0) | 1 | (1.0) | 2 | (2.6) | 3 | (1.9) |
| A*02:11 | 3 | (4.3) | 0 | (0.0) | 2 | (2.6) | 2 | (1.3) |
| A*02:12 | 1 | (1.4) | 1 | (1.0) | 2 | (2.6) | 2 | (1.3) |
| A*02:19 | 1 | (1.4) | 4 | (4.1) | 3 | (3.9) | 6 | (3.8) |
| A*02:22 | 1 | (1.4) | 4 | (4.1) | 3 | (3.9) | 6 | (3.8) |
| A*02:25 | 2 | (2.9) | 0 | (0.0) | 0 | (0.0) | 0 | (0.0) |
| A*02:34 | 0 | (0.0) | 3 | (3.1) | 0 | (0.0) | 3 | (1.9) |
| A*02:36 | 3 | (4.3) | 3 | (3.1) | 1 | (1.3) | 3 | (1.9) |
| A*02:37 | 0 | (0.0) | 1 | (1.0) | 2 | (2.6) | 3 | (1.9) |
| A*02:39 | 1 | (1.4) | 0 | (0.0) | 1 | (1.3) | 1 | (0.6) |
| A*02:40 | 0 | (0.0) | 4 | (4.1) | 1 | (1.3) | 5 | (3.2) |
| A*02:45 | 0 | (0.0) | 1 | (1.0) | 1 | (1.3) | 1 | (0.6) |
| A*02:46 | 0 | (0.0) | 1 | (1.0) | 0 | (0.0) | 1 | (0.6) |
| A*02:50 | 0 | (0.0) | 0 | (0.0) | 1 | (1.3) | 1 | (0.6) |
| A*02:56 | 1 | (1.4) | 1 | (1.0) | 0 | (0.0) | 1 | (0.6) |
| A*02:63 | 0 | (0.0) | 0 | (0.0) | 1 | (1.3) | 1 | (0.6) |
| A*02:65 | 1 | (1.4) | 0 | (0.0) | 0 | (0.0) | 0 | (0.0) |
| A*02:78 | 0 | (0.0) | 0 | (0.0) | 1 | (1.3) | 1 | (0.6) |
| A*02:80 | 0 | (0.0) | 0 | (0.0) | 1 | (1.3) | 1 | (0.6) |
| A*03:01 | 6 | (8.6) | 4 | (4.1) | 1 | (1.3) | 5 | (3.2) |
| A*03:02 | 0 | (0.0) | 1 | (1.0) | 2 | (2.6) | 3 | (1.9) |
| A*03:09 | 0 | (0.0) | 1 | (1.0) | 0 | (0.0) | 1 | (0.6) |
| A*03:23 | 0 | (0.0) | 1 | (1.0) | 0 | (0.0) | 1 | (0.6) |
| A*03:24 | 0 | (0.0) | 2 | (2.0) | 0 | (0.0) | 2 | (1.3) |
| A*11:01 | 2 | (2.9) | 6 | (6.1) | 2 | (2.6) | 7 | (4.4) |
| A*11:06 | 1 | (1.4) | 0 | (0.0) | 0 | (0.0) | 0 | (0.0) |
| A*11:08 | 0 | (0.0) | 1 | (1.0) | 0 | (0.0) | 1 | (0.6) |
| A*11:10 | 0 | (0.0) | 1 | (1.0) | 0 | (0.0) | 1 | (0.6) |
| A*11:19 | 0 | (0.0) | 1 | (1.0) | 0 | (0.0) | 1 | (0.6) |
| A*23:01 | 3 | (4.3) | 0 | (0.0) | 5 | (6.5) | 5 | (3.2) |
| A*23:02 | 0 | (0.0) | 1 | (1.0) | 0 | (0.0) | 1 | (0.6) |
| A*23:04 | 0 | (0.0) | 1 | (1.0) | 1 | (1.3) | 1 | (0.6) |
| A*24:02 | 18 | (25.7) | 21 | (21.4) | 13 | (16.9) | 31 | (19.6) |
| A*24:03 | 2 | (2.9) | 1 | (1.0) | 2 | (2.6) | 3 | (1.9) |
| A*24:06 | 0 | (0.0) | 2 | (2.0) | 1 | (1.3) | 2 | (1.3) |
| A*24:10 | 0 | (0.0) | 0 | (0.0) | 1 | (1.3) | 1 | (0.6) |
| A*24:14 | 5 | (7.1) | 4 | (4.1) | 1 | (1.3) | 4 | (2.5) |
| A*24:18 | 1 | (1.4) | 0 | (0.0) | 0 | (0.0) | 0 | (0.0) |
| A*24:50 | 1 | (1.4) | 0 | (0.0) | 0 | (0.0) | 0 | (0.0) |
| A*24:57 | 0 | (0.0) | 0 | (0.0) | 2 | (2.6) | 2 | (1.3) |
| A*24:62 | 0 | (0.0) | 1 | (1.0) | 0 | (0.0) | 1 | (0.6) |
| A*24:66 | 1 | (1.4) | 0 | (0.0) | 0 | (0.0) | 0 | (0.0) |
| A*25:01 | 0 | (0.0) | 1 | (1.0) | 0 | (0.0) | 1 | (0.6) |
| A*26:01 | 2 | (2.9) | 1 | (1.0) | 2 | (2.6) | 2 | (1.3) |
| A*26:02 | 0 | (0.0) | 1 | (1.0) | 0 | (0.0) | 1 | (0.6) |
| A*26:11 | 0 | (0.0) | 4 | (4.1) | 0 | (0.0) | 4 | (2.5) |
| A*26:18 | 0 | (0.0) | 1 | (1.0) | 0 | (0.0) | 1 | (0.6) |
| A*29:01 | 2 | (2.9) | 4 | (4.1) | 2 | (2.6) | 5 | (3.2) |
| A*29:02 | 0 | (0.0) | 0 | (0.0) | 1 | (1.3) | 1 | (0.6) |
| A*29:03 | 1 | (1.4) | 0 | (0.0) | 0 | (0.0) | 0 | (0.0) |
| A*29:09 | 2 | (2.9) | 4 | (4.1) | 2 | (2.6) | 6 | (3.8) |
| A*30:01 | 1 | (1.4) | 2 | (2.0) | 1 | (1.3) | 3 | (1.9) |
| A*30:02 | 2 | (2.9) | 2 | (2.0) | 2 | (2.6) | 3 | (1.9) |
| A*30:04 | 1 | (1.4) | 5 | (5.1) | 2 | (2.6) | 6 | (3.8) |
| A*30:09 | 0 | (0.0) | 0 | (0.0) | 1 | (1.3) | 1 | (0.6) |
| A*30:16 | 1 | (1.4) | 0 | (0.0) | 0 | (0.0) | 0 | (0.0) |
| A*30:17 | 1 | (1.4) | 1 | (1.0) | 0 | (0.0) | 1 | (0.6) |
| A*31:01 | 4 | (5.7) | 11 | (11.2) | 11 | (14.3) | 18 | (11.4) |
| A*31:07 | 2 | (2.9) | 1 | (1.0) | 3 | (3.9) | 4 | (2.5) |
| A*31:08 | 0 | (0.0) | 1 | (1.0) | 0 | (0.0) | 1 | (0.6) |
| A*31:12 | 1 | (1.4) | 0 | (0.0) | 0 | (0.0) | 0 | (0.0) |
| A*31:17 | 1 | (1.4) | 0 | (0.0) | 0 | (0.0) | 0 | (0.0) |
| A*32:01 | 0 | (0.0) | 0 | (0.0) | 1 | (1.3) | 1 | (0.6) |
| A*33:01 | 1 | (1.4) | 5 | (5.1) | 1 | (1.3) | 6 | (3.8) |
| A*34:02 | 2 | (2.9) | 0 | (0.0) | 0 | (0.0) | 0 | (0.0) |
| A*66:01 | 0 | (0.0) | 0 | (0.0) | 1 | (1.3) | 1 | (0.6) |
| A*68:01 | 23 | (32.9) | 22 | (22.4) | 15 | (19.5) | 32 | (20.3) |
| A*68:02 | 2 | (2.9) | 0 | (0.0) | 1 | (1.3) | 1 | (0.6) |
| A*68:03 | 0 | (0.0) | 3 | (3.1) | 0 | (0.0) | 3 | (1.9) |
| A*68:06 | 0 | (0.0) | 1 | (1.0) | 0 | (0.0) | 1 | (0.6) |
| A*68:19 | 0 | (0.0) | 2 | (2.0) | 0 | (0.0) | 2 | (1.3) |
| A*68:23 | 2 | (2.9) | 0 | (0.0) | 0 | (0.0) | 0 | (0.0) |
| A*68:26 | 0 | (0.0) | 1 | (1.0) | 0 | (0.0) | 1 | (0.6) |
| A*68:27 | 0 | (0.0) | 0 | (0.0) | 1 | (1.3) | 1 | (0.6) |
| A*74:03 | 0 | (0.0) | 0 | (0.0) | 1 | (1.3) | 1 | (0.6) |
| A*74:04 | 1 | (1.4) | 0 | (0.0) | 0 | (0.0) | 0 | (0.0) |
