# Supplementary material for: Protective Human Leucocyte Antigen Haplotype, HLA-DRB1*01-B*14, against Chronic Chagas Disease in Bolivia
Source: PLoS Negl Trop Dis. 2012 Mar 20;6(3):e1587. doi: 10.1371/journal.pntd.0001587 (PMC3308929; doi:10.1371/journal.pntd.0001587)
Supplement: Table S5 — The frequency of the Alleles of HLA-B locus. Two digits analysis. (DOC) [file pntd.0001587.s005.doc]

**Table S5. The frequency of the Alleles of HLA-B locus. Two digits analysis**

|  | **Indeterminate**  **(N=70)** | | **Megacolon**  **(N=98)** | | **ECG**  **Alteration**  **(N=77)** | | **ECG alteration and/or Megacolon (N=158)** | |
| --- | --- | --- | --- | --- | --- | --- | --- | --- |
|  | n | (%) | n | (%) | n | (%) | n | (%) |
| B*07 | 6 | (8.6) | 11 | (11.2) | 7 | (9.1) | 18 | (11.4) |
| B*08 | 8 | (11.4) | 6 | (6.1) | 6 | (7.8) | 12 | (7.6) |
| B*13 | 1 | (1.4) | 1 | (1.0) | 1 | (1.3) | 1 | (0.6) |
| B*14 | 10 | (14.3) | 2 | (2.0) | 3 | (3.9) | 5 | (3.2) |
| B*15 | 7 | (10.0) | 18 | (18.4) | 13 | (16.9) | 29 | (18.4) |
| B*18 | 5 | (7.1) | 3 | (3.1) | 0 | (0.0) | 3 | (1.9) |
| B*27 | 1 | (1.4) | 1 | (1.0) | 2 | (2.6) | 3 | (1.9) |
| B*35 | 33 | (47.1) | 41 | (41.8) | 41 | (53.2) | 71 | (44.9) |
| B*37 | 1 | (1.4) | 0 | (0.0) | 1 | (1.3) | 1 | (0.6) |
| B*38 | 5 | (7.1) | 1 | (1.0) | 2 | (2.6) | 3 | (1.9) |
| B*39 | 8 | (11.4) | 19 | (19.4) | 12 | (15.6) | 28 | (17.7) |
| B*40 | 9 | (12.9) | 15 | (15.3) | 10 | (13.0) | 22 | (13.9) |
| B*41 | 0 | (0.0) | 1 | (1.0) | 3 | (3.9) | 3 | (1.9) |
| B*42 | 0 | (0.0) | 1 | (1.0) | 0 | (0.0) | 1 | (0.6) |
| B*44 | 11 | (15.7) | 9 | (9.2) | 8 | (10.4) | 16 | (10.1) |
| B*45 | 2 | (2.9) | 2 | (2.0) | 0 | (0.0) | 2 | (1.3) |
| B*48 | 10 | (14.3) | 17 | (17.3) | 9 | (11.7) | 23 | (14.6) |
| B*49 | 2 | (2.9) | 3 | (3.1) | 2 | (2.6) | 4 | (2.5) |
| B*50 | 1 | (1.4) | 0 | (0.0) | 1 | (1.3) | 1 | (0.6) |
| B*51 | 10 | (14.3 | 19 | (19.4 | 13 | (16.9) | 30 | (19.0) |
| B*52 | 2 | (2.9) | 3 | (3.1) | 3 | (3.9) | 5 | (3.2) |
| B*53 | 3 | (4.3) | 3 | (3.1) | 6 | (7.8) | 7 | (4.4) |
| B*55 | 0 | (0.0) | 1 | (1.0) | 0 | (0.0) | 1 | (0.6) |
| B*56 | 0 | (0.0) | 0 | (0.0) | 0 | (0.0) | 0 | (0.0) |
| B*57 | 1 | (1.4) | 1 | (1.0) | 1 | (1.3) | 2 | (1.3) |
| B*58 | 1 | (1.4) | 2 | (2.0) | 1 | (1.3) | 3 | (1.9) |
| B*67 | 1 | (1.4) | 0 | (0.0) | 0 | (0.0) | 0 | (0.0) |
| B*78 | 1 | (1.4) | 0 | (0.0) | 1 | (1.3) | 1 | (0.6) |
