# Supplementary material for: Protective Human Leucocyte Antigen Haplotype, HLA-DRB1*01-B*14, against Chronic Chagas Disease in Bolivia
Source: PLoS Negl Trop Dis. 2012 Mar 20;6(3):e1587. doi: 10.1371/journal.pntd.0001587 (PMC3308929; doi:10.1371/journal.pntd.0001587)
Supplement: Table S6 — The frequency of the Alleles of HLA-B locus. Four digits analysis. (DOC) [file pntd.0001587.s006.doc]

**Table S6.** The frequency of the Alleles of HLA-B locus. Four digits analysis

|  | **Indeterminate**  **(N=70)** | | **Megacolon**  **(N=98)** | | **ECG**  **Alteration**  **(N=77)** | | **ECG alteration and/or Megacolon (N=158)** | |
| --- | --- | --- | --- | --- | --- | --- | --- | --- |
|  | n | (%) | n | (%) | n | (%) | n | (%) |
| B*08:01 | 4 | (5.7) | 6 | (6.1) | 5 | (6.5) | 11 | (7.0) |
| B*07:02 | 3 | (4.3) | 9 | (9.2) | 4 | (5.2) | 13 | (8.2) |
| B*07:04 | 1 | (1.4) | 0 | (0.0) | 0 | (0.0) | 0 | (0.0) |
| B*07:05 | 1 | (1.4) | 0 | (0.0) | 0 | (0.0) | 0 | (0.0) |
| B*07:08 | 1 | (1.4) | 0 | (0.0) | 0 | (0.0) | 0 | (0.0) |
| B*07:12 | 0 | (0.0) | 1 | (1.0) | 0 | (0.0) | 1 | (0.6) |
| B*07:27 | 0 | (0.0) | 0 | (0.0) | 1 | (1.3) | 1 | (0.6) |
| B*07:41 | 0 | (0.0) | 1 | (1.0) | 0 | (0.0) | 1 | (0.6) |
| B*07:48 | 0 | (0.0) | 0 | (0.0) | 1 | (1.3) | 1 | (0.6) |
| B*07:50 | 0 | (0.0) | 0 | (0.0) | 1 | (1.3) | 1 | (0.6) |
| B*07:53 | 0 | (0.0) | 0 | (0.0) | 0 | (0.0) | 0 | (0.0) |
| B*08:01 | 4 | (5.7) | 6 | (6.1) | 5 | (6.5) | 11 | (7.0) |
| B*08:02 | 1 | (1.4) | 0 | (0.0) | 0 | (0.0) | 0 | (0.0) |
| B*08:03 | 1 | (1.4) | 0 | (0.0) | 0 | (0.0) | 0 | (0.0) |
| B*08:12 | 0 | (0.0) | 0 | (0.0) | 1 | (1.3) | 1 | (0.6) |
| B*08:13 | 1 | (1.4) | 0 | (0.0) | 0 | (0.0) | 0 | (0.0) |
| B*08:21 | 1 | (1.4) | 0 | (0.0) | 0 | (0.0) | 0 | (0.0) |
| B*13:02 | 0 | (0.0) | 1 | (1.0) | 1 | (1.3) | 1 | (0.6) |
| B*13:09 | 1 | (1.4) | 0 | (0.0) | 0 | (0.0) | 0 | (0.0) |
| B*14:01 | 1 | (1.4) | 0 | (0.0) | 2 | (2.6) | 2 | (1.3) |
| B*14:02 | 8 | (11.4) | 2 | (2.0) | 1 | (1.3) | 3 | (1.9) |
| B*14:06 | 1 | (1.4) | 0 | (0.0) | 0 | (0.0) | 0 | (0.0) |
| B*15:01 | 1 | (1.4) | 5 | (5.1) | 3 | (3.9) | 7 | (4.4) |
| B*15:03 | 0 | (0.0) | 0 | (0.0) | 1 | (1.3) | 1 | (0.6) |
| B*15:04 | 3 | (4.3) | 5 | (5.1) | 3 | (3.9) | 8 | (5.1) |
| B*15:07 | 0 | (0.0) | 2 | (2.0) | 0 | (0.0) | 2 | (1.3) |
| B*15:10 | 0 | (0.0) | 0 | (0.0) | 1 | (1.3) | 1 | (0.6) |
| B*15:15 | 0 | (0.0) | 1 | (1.0) | 0 | (0.0) | 1 | (0.6) |
| B*15:16 | 0 | (0.0) | 0 | (0.0) | 1 | (1.3) | 1 | (0.6) |
| B*15:20 | 1 | (1.4) | 1 | (1.0) | 0 | (0.0) | 1 | (0.6) |
| B*15:26 | 0 | (0.0) | 2 | (2.0) | 1 | (1.3) | 2 | (1.3) |
| B*15:30 | 0 | (0.0) | 1 | (1.0) | 0 | (0.0) | 1 | (0.6) |
| B*15:38 | 0 | (0.0) | 0 | (0.0) | 1 | (1.3) | 1 | (0.6) |
| B*15:39 | 0 | (0.0) | 0 | (0.0) | 1 | (1.3) | 1 | (0.6) |
| B*15:54 | 0 | (0.0) | 1 | (1.0) | 0 | (0.0) | 1 | (0.6) |
| B*15:69 | 0 | (0.0) | 0 | (0.0) | 1 | (1.3) | 1 | (0.6) |
| B*15:70 | 1 | (1.4) | 0 | (0.0) | 0 | (0.0) | 0 | (0.0) |
| B*15:85 | 1 | (1.4) | 0 | (0.0) | 0 | (0.0) | 0 | (0.0) |
| B*18:01 | 2 | (2.9) | 3 | (3.1) | 0 | (0.0) | 3 | (1.9) |
| B*18:04 | 1 | (1.4) | 0 | (0.0) | 0 | (0.0) | 0 | (0.0) |
| B*18:12 | 2 | (2.9) | 0 | (0.0) | 0 | (0.0) | 0 | (0.0) |
| B*27:02 | 0 | (0.0) | 1 | (1.0) | 0 | (0.0) | 1 | (0.6) |
| B*27:03 | 1 | (1.4) | 0 | (0.0) | 1 | (1.3) | 1 | (0.6) |
| B*27:30 | 0 | (0.0) | 0 | (0.0) | 1 | (1.3) | 1 | (0.6) |
| B*35:01 | 12 | (17.1) | 16 | (16.3) | 15 | (19.5) | 28 | (17.7) |
| B*35:02 | 1 | (1.4) | 0 | (0.0) | 0 | (0.0) | 0 | (0.0) |
| B*35:04 | 3 | (4.3) | 2 | (2.0) | 4 | (5.2) | 5 | (3.2) |
| B*35:05 | 7 | (10.0) | 12 | (12.2) | 12 | (15.6) | 21 | (13.3) |
| B*35:06 | 0 | (0.0) | 5 | (5.1) | 0 | (0.0) | 5 | (3.2) |
| B*35:08 | 0 | (0.0) | 0 | (0.0) | 1 | (1.3) | 1 | (0.6) |
| B*35:15 | 0 | (0.0) | 2 | (2.0) | 1 | (1.3) | 2 | (1.3) |
| B*35:19 | 0 | (0.0) | 2 | (2.0) | 1 | (1.3) | 2 | (1.3) |
| B*35:20 | 1 | (1.4) | 1 | (1.0) | 2 | (2.6) | 2 | (1.3) |
| B*35:21 | 4 | (5.7) | 2 | (2.0) | 2 | (2.6) | 4 | (2.5) |
| B*35:22 | 0 | (0.0) | 3 | (3.1) | 2 | (2.6) | 4 | (2.5) |
| B*35:24 | 1 | (1.4) | 0 | (0.0) | 0 | (0.0) | 0 | (0.0) |
| B*35:26 | 1 | (1.4) | 1 | (1.0) | 1 | (1.3) | 2 | (1.3) |
| B*35:28 | 0 | (0.0) | 0 | (0.0) | 2 | (2.6) | 2 | (1.3) |
| B*35:35 | 0 | (0.0) | 3 | (3.1) | 1 | (1.3) | 3 | (1.9) |
| B*35:43 | 0 | (0.0) | 2 | (2.0) | 2 | (2.6) | 3 | (1.9) |
| B*35:44 | 1 | (1.4) | 1 | (1.0) | 0 | (0.0) | 1 | (0.6) |
| B*35:58 | 0 | (0.0) | 1 | 1.0 | 0 | (0.0) | 1 | (0.6) |
| B*35:59 | 1 | (1.4) | 0 | (0.0) | 0 | (0.0) | 0 | (0.0) |
| B*35:68 | 1 | (1.4) | 0 | (0.0) | 0 | (0.0) | 0 | (0.0) |
| B*35:72 | 0 | (0.0) | 0 | (0.0) | 1 | (1.3) | 1 | (0.6) |
| B*37:01 | 1 | (1.4) | 0 | (0.0) | 1 | (1.3) | 1 | (0.6) |
| B*37:11 | 0 | (0.0) | 0 | (0.0) | 0 | (0.0) | 0 | (0.0) |
| B*38:01 | 4 | (5.7) | 1 | (1.0) | 2 | (2.6) | 3 | (1.9) |
| B*38:02 | 1 | (1.4) | 0 | (0.0) | 0 | (0.0) | 0 | (0.0) |
| B*39:01 | 0 | (0.0) | 4 | (4.1) | 3 | (3.9) | 7 | (4.4) |
| B*39:02 | 0 | (0.0) | 1 | (1.0) | 0 | (0.0) | 1 | (0.6) |
| B*39:03 | 1 | (1.4) | 1 | (1.0) | 2 | (2.6) | 2 | (1.3) |
| B*39:04 | 1 | (1.4) | 3 | (3.1) | 2 | (2.6) | 4 | (2.5) |
| B*39:05 | 0 | (0.0) | 1 | (1.0) | 3 | (3.9) | 4 | (2.5) |
| B*39:06 | 1 | (1.4) | 4 | (4.1) | 1 | (1.3) | 4 | (2.5) |
| B*39:08 | 0 | (0.0) | 0 | (0.0) | 0 | (0.0) | 0 | (0.0) |
| B*39:09 | 0 | (0.0) | 1 | (1.0) | 0 | (0.0) | 1 | (0.6) |
| B*39:10 | 0 | (0.0) | 1 | (1.0) | 0 | (0.0) | 1 | (0.6) |
| B*39:14 | 3 | (4.3) | 3 | (3.1) | 2 | (2.6) | 5 | (3.2) |
| B*39:35 | 1 | (1.4) | 0 | (0.0) | 0 | (0.0) | 0 | (0.0) |
| B*39:36 | 1 | (1.4) | 0 | (0.0) | 0 | (0.0) | 0 | (0.0) |
| B*39:37 | 0 | (0.0) | 1 | (1.0) | 0 | (0.0) | 1 | (0.6) |
| B*40:01 | 0 | (0.0) | 1 | (1.0) | 0 | (0.0) | 1 | (0.6) |
| B*40:02 | 2 | (2.9) | 9 | (9.2) | 4 | (5.2) | 11 | (7.0) |
| B*40:03 | 0 | (0.0) | 0 | (0.0) | 1 | (1.3) | 1 | (0.6) |
| B*40:04 | 1 | (1.4) | 1 | (1.0) | 2 | (2.6) | 2 | (1.3) |
| B*40:06 | 2 | (2.9) | 1 | (1.0) | 1 | (1.3) | 2 | (1.3) |
| B*40:08 | 0 | (0.0) | 1 | (1.0) | 0 | (0.0) | 1 | (0.6) |
| B*40:10 | 1 | (1.4) | 0 | (0.0) | 0 | (0.0) | 0 | (0.0) |
| B*40:12 | 0 | (0.0) | 1 | (1.0) | 0 | (0.0) | 1 | (0.6) |
| B*40:24 | 1 | (1.4) | 0 | (0.0) | 0 | (0.0) | 0 | (0.0) |
| B*40:28 | 0 | (0.0) | 1 | (1.0) | 0 | (0.0) | 1 | (0.6) |
| B*40:29 | 1 | (1.4) | 0 | (0.0) | 0 | (0.0) | 0 | (0.0) |
| B*40:32 | 1 | (1.4) | 0 | (0.0) | 0 | (0.0) | 0 | (0.0) |
| B*40:40 | 0 | (0.0) | 0 | (0.0) | 1 | (1.3) | 1 | (0.6) |
| B*40:46 | 0 | (0.0) | 1 | (1.0) | 0 | (0.0) | 1 | (0.6) |
| B*40:52 | 0 | (0.0) | 0 | (0.0) | 1 | (1.3) | 1 | (0.6) |
| B*41:01 | 0 | (0.0) | 1 | (1.0) | 3 | (3.9) | 3 | (1.9) |
| B*42:01 | 0 | (0.0) | 1 | (1.0) | 0 | (0.0) | 1 | (0.6) |
| B*44:02 | 1 | (1.4) | 1 | (1.0) | 0 | (0.0) | 1 | (0.6) |
| B*44:03 | 5 | (7.1) | 5 | (5.1) | 4 | (5.2) | 8 | (5.1) |
| B*44:04 | 0 | (0.0) | 1 | (1.0) | 0 | (0.0) | 1 | (0.6) |
| B*44:09 | 1 | (1.4) | 1 | (1.0) | 2 | (2.6) | 3 | (1.9) |
| B*44:12 | 1 | (1.4) | 0 | (0.0) | 0 | (0.0) | 0 | (0.0) |
| B*44:18 | 1 | (1.4) | 0 | (0.0) | 0 | (0.0) | 0 | (0.0) |
| B*44:46 | 2 | (2.9) | 0 | (0.0) | 1 | (1.3) | 1 | (0.6) |
| B*44:47 | 0 | (0.0) | 1 | (1.0) | 0 | (0.0) | 1 | (0.6) |
| B*44:54 | 0 | (0.0) | 0 | (0.0) | 1 | (1.3) | 1 | (0.6) |
| B*45:01 | 2 | (2.9) | 1 | (1.0) | 0 | (0.0) | 1 | (0.6) |
| B*45:02 | 0 | (0.0) | 1 | (1.0) | 0 | (0.0) | 1 | (0.6) |
| B*48:01 | 7 | (10.0) | 9 | (9.2) | 6 | (7.8) | 14 | (8.9) |
| B*48:02 | 0 | (0.0) | 1 | (1.0) | 0 | (0.0) | 1 | (0.6) |
| B*48:03 | 2 | (2.9) | 3 | (3.1) | 0 | (0.0) | 3 | (1.9) |
| B*48:15 | 1 | (1.4) | 4 | (4.1) | 3 | (3.9) | 5 | (3.2) |
| B*49:01 | 0 | (0.0) | 3 | (3.1) | 1 | (1.3) | 3 | (1.9) |
| B*49:02 | 1 | (1.4) | 0 | (0.0) | 0 | (0.0) | 0 | (0.0) |
| B*49:04 | 1 | (1.4) | 0 | (0.0) | 1 | (1.3) | 1 | (0.6) |
| B*50:01 | 1 | (1.4) | 0 | (0.0) | 1 | (1.3) | 0 | (0.0) |
| B*50:04 | 0 | (0.0) | 0 | (0.0) | 0 | (0.0) | 1 | (0.6) |
| B*51:01 | 3 | (4.3) | 9 | (9.2) | 8 | (10.4) | 16 | (10.1) |
| B*51:02 | 2 | (2.9) | 0 | (0.0) | 0 | (0.0) | 0 | (0.0) |
| B*51:06 | 1 | (1.4) | 0 | (0.0) | 1 | (1.3) | 0 | (0.0) |
| B*51:08 | 0 | (0.0) | 1 | (1.0) | 1 | (1.3) | 1 | (0.6) |
| B*51:09 | 0 | (0.0) | 1 | (1.0) | 0 | (0.0) | 2 | (1.3) |
| B*51:11 | 0 | (0.0) | 1 | (1.0) | 0 | (0.0) | 1 | (0.6) |
| B*51:13 | 2 | (2.9) | 6 | (6.1) | 2 | (2.6) | 1 | (0.6) |
| B*51:21 | 1 | (1.4) | 0 | (0.0) | 1 | (1.3) | 7 | (4.4) |
| B*51:31 | 1 | (1.4) | 0 | (0.0) | 0 | (0.0) | 1 | (0.6) |
| B*51:37 | 0 | (0.0) | 0 | (0.0) | 0 | (0.0) | 0 | (0.0) |
| B*51:47 | 0 | (0.0) | 1 | (1.0) | 0 | (0.0) | 0 | (0.0) |
| B*52:01 | 2 | (2.9) | 3 | (3.1) | 3 | (3.9) | 1 | (0.6) |
| B*53:01 | 2 | (2.9) | 1 | (1.0) | 2 | (2.6) | 5 | (3.2) |
| B*53:02 | 0 | (0.0) | 0 | (0.0) | 1 | (1.3) | 3 | (1.9) |
| B*53:05 | 0 | (0.0) | 0 | (0.0) | 1 | (1.3) | 1 | (0.6) |
| B*53:13 | 1 | (1.4) | 2 | (2.0) | 2 | (2.6) | 1 | (0.6) |
| B*55:01 | 0 | (0.0) | 1 | (1.0) | 0 | (0.0) | 2 | (1.3) |
| B*56:01 | 0 | (0.0) | 0 | (0.0) | 0 | (0.0) | 1 | (0.6) |
| B*57:01 | 1 | (1.4) | 0 | (0.0) | 1 | (1.3) | 0 | (0.0) |
| B*57:10 | 0 | (0.0) | 1 | (1.0) | 0 | (0.0) | 1 | (0.6) |
| B*58:01 | 1 | (1.4) | 1 | (1.0) | 1 | (1.3) | 1 | (0.6) |
| B*58:02 | 0 | (0.0) | 1 | (1.0) | 0 | (0.0) | 2 | (1.3) |
| B*59:01 | 0 | (0.0) | 0 | (0.0) | 0 | (0.0) | 1 | (0.6) |
| B*67:01 | 1 | (1.4) | 0 | (0.0) | 0 | (0.0) | 0 | (0.0) |
| B*78:02 | 1 | (1.4) | 0 | (0.0) | 1 | (1.3) | 0 | (0.0) |
| B*78:05 | 0 | (0.0) | 0 | (0.0) | 0 | (0.0) | 1 | (0.6) |
