# Supplementary material for: Protective Human Leucocyte Antigen Haplotype, HLA-DRB1*01-B*14, against Chronic Chagas Disease in Bolivia
Source: PLoS Negl Trop Dis. 2012 Mar 20;6(3):e1587. doi: 10.1371/journal.pntd.0001587 (PMC3308929; doi:10.1371/journal.pntd.0001587)
Supplement: Table S7 — The frequency of the Alleles of MICA locus. (DOC) [file pntd.0001587.s007.doc]

**Table S7.** The frequency of the Alleles of MICA locus

|  | **Indeterminate**  **(N=70)** | | **Megacolon**  **(N=98)** | | **ECG**  **Alteration**  **(N=77)** | | **ECG alteration and/or Megacolon (N=158)** | |
| --- | --- | --- | --- | --- | --- | --- | --- | --- |
|  | n | (%) | n | (%) | n | (%) | n | (%) |
| MICA*001 | 3 | (4.3) | 0 | (0.0) | 0 | (0.0) | 0 | (0.0) |
| MICA*002 | 5 | (7.1) | 9 | (9.2) | 7 | (9.1) | 13 | (8.2) |
| MICA*004 | 5 | (7.1) | 9 | (9.2) | 7 | (9.1) | 13 | (8.2) |
| MICA*005 | 2 | (2.9) | 3 | (3.1) | 5 | (6.5) | 6 | (3.8) |
| MICA*006 | 1 | (1.4) | 0 | (0.0) | 0 | (0.0) | 0 | (0.0) |
| MICA*007 | 4 | (5.7) | 7 | (7.1) | 5 | (6.5) | 11 | (7.0) |
| MICA*008 | 16 | (22.9) | 21 | (21.4) | 17 | (22.1) | 33 | (20.9) |
| MICA*009 | 11 | (15.7) | 17 | (17.3) | 12 | (15.6) | 26 | (16.5) |
| MICA*010 | 0 | (0.0) | 5 | (5.1) | 3 | (3.9) | 8 | (5.1) |
| MICA*011 | 6 | (8.6) | 0 | (0.0) | 1 | (1.3) | 1 | (0.6) |
| MICA*012 | 1 | (1.4) | 0 | (0.0) | 0 | (0.0) | 0 | (0.0) |
| MICA*017 | 1 | (1.4) | 0 | (0.0) | 0 | (0.0) | 0 | (0.0) |
| MICA*018 | 0 | (0.0) | 0 | (0.0) | 2 | (2.6) | 2 | (1.3) |
| MICA*019 | 7 | (10.0) | 12 | (12.2) | 5 | (6.5) | 16 | (10.1) |
| MICA*022 | 3 | (4.3) | 2 | (2.0) | 2 | (2.6) | 3 | (1.9) |
| MICA*023 | 8 | (11.4) | 13 | (13.3) | 17 | (22.1) | 26 | (16.5) |
| MICA*024 | 19 | (27.1) | 19 | 19.4) | 15 | (19.5) | 32 | (20.3) |
| MICA*025 | 1 | (1.4) | 0 | (0.0) | 1 | (1.3) | 1 | (0.6) |
| MICA*030 | 1 | (1.4) | 2 | (2.0) | 2 | (2.6) | 4 | (2.5) |
| MICA*034 | 1 | (1.4) | 4 | (4.1) | 2 | (2.6) | 6 | (3.8) |
| MICA*035 | 4 | (5.7) | 10 | (10.2) | 6 | (7.8) | 15 | (9.5) |
| MICA*037 | 1 | (1.4) | 2 | (2.0) | 1 | (1.3) | 3 | (1.9) |
| MICA*038 | 15 | (21.4) | 17 | (17.3) | 8 | (10.4) | 25 | (15.8) |
| MICA*040 | 1 | (1.4) | 1 | (1.0) | 0 | (0.0) | 1 | (0.6) |
| MICA*041 | 15 | (21.4) | 23 | (23.5) | 19 | (24.7) | 38 | (24.1) |
| MICA*044 | 1 | (1.4) | 2 | (2.0) | 4 | (5.2) | 4 | (2.5) |
| MICA*050 | 0 | (0.0) | 3 | (3.1) | 1 | (1.3) | 3 | (1.9) |
| MICA*053 | 1 | (1.4) | 2 | (2.0) | 0 | (0.0) | 2 | (1.3) |
| MICA*054 | 0 | (0.0) | 0 | (0.0) | 1 | (1.3) | 1 | (0.6) |
| MICA*057 | 1 | (1.4) | 4 | (4.1) | 2 | (2.6) | 5 | (3.2) |
| Not Identified | 1 | (1.4) | 4 | (4.1) | 3 | (3.9) | 7 | (4.4) |
