# Supplementary material for: Protective Human Leucocyte Antigen Haplotype, HLA-DRB1*01-B*14, against Chronic Chagas Disease in Bolivia
Source: PLoS Negl Trop Dis. 2012 Mar 20;6(3):e1587. doi: 10.1371/journal.pntd.0001587 (PMC3308929; doi:10.1371/journal.pntd.0001587)
Supplement: Table S8 — The frequency of the Alleles of MICB locus. (DOC) [file pntd.0001587.s008.doc]

**Table S8.** The frequency of the Alleles of MICB locus

|  | **Indeterminate**  **(N=70)** | | **Megacolon**  **(N=98)** | | **ECG**  **Alteration**  **(N=77)** | | **ECG alteration and/or Megacolon (N=158)** | |
| --- | --- | --- | --- | --- | --- | --- | --- | --- |
|  | n | (%) | n | (%) | n | (%) | n | (%) |
| MICB*002 | 10 | (14.3) | 23 | (23.5) | 9 | (11.7) | 31 | (19.6) |
| MICB*003 | 2 | (2.9) | 6 | (6.1) | 5 | (6.5) | 11 | (7.0) |
| MICB*004 | 15 | (21.4) | 23 | (23.5) | 18 | (23.4) | 37 | (23.4) |
| MICB*005 | 59 | (84.3) | 80 | (81.6) | 62 | (80.5) | 125 | (79.1) |
| MICB*007 | 0 | (0.0) | 1 | (1.0) | 1 | (1.3) | 1 | (0.6) |
| MICB*008 | 9 | (12.9) | 5 | (5.1) | 9 | (11.7) | 14 | (8.9) |
| MICB*009 | 5 | (7.1) | 11 | (11.2) | 9 | (11.7) | 18 | (11.4) |
| MICB*013 | 0 | (0.0) | 1 | (1.0) | 0 | (0.0) | 1 | (0.6) |
| MICB*014 | 0 | (0.0) | 1 | (1.0) | 0 | (0.0) | 1 | (0.6) |
| MICB*018 | 3 | (4.3) | 4 | (4.1) | 1 | (1.3) | 4 | (2.5) |
| MICB*019 | 4 | (5.7) | 2 | (2.0) | 2 | (2.6) | 4 | (2.5) |
| Not Identified | 1 | (1.4) | 1 | (1.0) | 0 | (0.0) | 1 | (0.6) |
