# Supplementary material for: Protective Human Leucocyte Antigen Haplotype, HLA-DRB1*01-B*14, against Chronic Chagas Disease in Bolivia
Source: PLoS Negl Trop Dis. 2012 Mar 20;6(3):e1587. doi: 10.1371/journal.pntd.0001587 (PMC3308929; doi:10.1371/journal.pntd.0001587)
Supplement: Table S9 — The frequency of GCT triplet polymorphism in the MICA-transmembrane region. (DOC) [file pntd.0001587.s009.doc]

**Table S9.** The frequency of GCT triplet polymorphism in the MICA-transmembrane region

|  | **Indeterminate**  **(N=70)** | | **Megacolon**  **(N=98)** | | **ECG**  **Alteration**  **(N=77)** | | **ECG alteration and/or Megacolon (N=158)** | |
| --- | --- | --- | --- | --- | --- | --- | --- | --- |
|  | n | (%) | n | (%) | n | (%) | n | (%) |
| A4 | 7 | (10.0) | 5 | (5.1) | 4 | (5.2) | 9 | (5.7) |
| A5 | 26 | (37.1) | 43 | (43.9) | 26 | (33.8) | 63 | (39.9) |
| A5.1 | 16 | (22.9) | 18 | (18.4) | 20 | (26.0) | 36 | (22.8) |
| A6 | 24 | (34.3) | 27 | (27.6) | 21 | (27.3) | 41 | (25.9) |
| A9 | 44 | (62.9) | 59 | (60.2) | 54 | (70.1) | 101 | (63.9) |
| Not Identified | 1 | (1.4) | 2 | (2.0) | 1 | (1.3) | 3 | (1.9) |
