# Supplementary material for: Protective Human Leucocyte Antigen Haplotype, HLA-DRB1*01-B*14, against Chronic Chagas Disease in Bolivia
Source: PLoS Negl Trop Dis. 2012 Mar 20;6(3):e1587. doi: 10.1371/journal.pntd.0001587 (PMC3308929; doi:10.1371/journal.pntd.0001587)
Supplement: Table S10 — The frequency of B Supertype. (DOC) [file pntd.0001587.s010.doc]

**Table S10.** The frequency of B Supertype

|  | **Indeterminate**  **(N=70)** | | **Megacolon**  **(N=98)** | | **ECG**  **Alteration**  **(N=77)** | | **ECG alteration and/or Megacolon (N=158)** | |
| --- | --- | --- | --- | --- | --- | --- | --- | --- |
|  | n | (%) | n | (%) | n | (%) | n | (%) |
| B07 | 42 | (60.0) | 63 | (64.3) | 51 | (66.2) | 103 | (65.2) |
| B08 | 8 | (11.4) | 6 | (6.1) | 6 | (7.8) | 12 | (7.6) |
| B27 | 30 | (42.9) | 34 | (34.7) | 27 | (35.1) | 57 | (36.1) |
| B44 | 18 | (25.7) | 24 | (24.5) | 19 | (24.7) | 38 | (24.1) |
| B58 | 2 | (2.9) | 2 | (2.0) | 3 | (3.9) | 5 | (3.2) |
| B62 | 9 | (12.9) | 18 | (18.4) | 13 | (16.9) | 29 | (18.4) |
| Not Identified | 18 | (25.7) | 23 | (23.5) | 19 | (24.7) | 33 | (20.9) |
