# Supplementary material for: Protective Human Leucocyte Antigen Haplotype, HLA-DRB1*01-B*14, against Chronic Chagas Disease in Bolivia
Source: PLoS Negl Trop Dis. 2012 Mar 20;6(3):e1587. doi: 10.1371/journal.pntd.0001587 (PMC3308929; doi:10.1371/journal.pntd.0001587)
Supplement: Table S11 — The frequency of TNF-alpha promoter region polymorphism. (DOC) [file pntd.0001587.s011.doc]

**Table S11.** The frequency of TNF-alpha promoter region polymorphism

|  | **Indeterminate**  **(N=70)** | | **Megacolon**  **(N=98)** | | **ECG**  **Alteration**  **(N=77)** | | **ECG alteration and/or Megacolon (N=158)** | |
| --- | --- | --- | --- | --- | --- | --- | --- | --- |
|  | n | (%) | n | (%) | n | (%) | n | (%) |
| TNFA | 44 | (62.9) | 67 | (68.4) | 55 | (71.4) | 107 | (67.7) |
| TNFB | 16 | (22.9) | 17 | (17.3) | 11 | (14.3) | 26 | (16.5) |
| TNFC | 9 | (12.9) | 14 | (14.3) | 10 | (13.0) | 22 | (13.9) |
| TNFD | 31 | (44.3) | 39 | (39.8) | 35 | (45.5) | 67 | (42.4) |
| TNFE | 7 | (10.0) | 6 | (6.1) | 7 | (9.1) | 13 | (8.2) |
| TNFF | 1 | (1.4) | 0 | (0.0) | 1 | (1.3) | 1 | (0.6) |
| Not Identified | 2 | (2.9) | 7 | (6.1) | 3 | (1.3) | 10 | (4.4) |
